# Neuroinflammation Screening in Immunotherapy Trials against Alzheimer's Disease

**DOI:** 10.4061/2010/638379

**Published:** 2010-12-19

**Authors:** Niels Andreasen, Kaj Blennow, Henrik Zetterberg

**Affiliations:** ^1^Memory Clinic, M51, Department of Geriatric Medicine, Karolinska University Hospital, Huddinge, 17176 Stockholm, Sweden; ^2^Department of Psychiatry and Neurochemistry, Institute of Neuroscience and Physiology, The Sahlgrenska Academy at the University of Gothenburg, 431 80 Mölndal, Sweden

## Abstract

Due to side effects in the form of meningoencephalitis in the interrupted phase II AN1792 trial of active antiamyloid **β**(A**β**) immunization against Alzheimer's disease (AD), there has been concern that anti-A**β** immunization may cause destructive neuroinflammation. Here, we report on two patients fulfilling clinical AD criteria who were diagnosed with Lyme neuroborreliosis during screening before inclusion in anti-A**β** immunotherapy trials. The two cases illustrate the necessity of careful biochemical screening for neuroinflammatory/neuroinfectious conditions before an AD diagnosis is made and before clinical AD patients are included in trials of therapy that could impact the immune system. Should the two cases have been included and deteriorated, additional investigations might have led to the erroneous conclusion that therapy-induced meningoencephalitis had occurred.

## 1. Introduction

Clinical trials of disease-modifying treatments for AD have been struck by adverse events involving the immune system. In the clinical trial of the active antiamyloid *β*(A*β*) vaccine AN1792, a small but notable number of patients developed meningoencephalitis [1], and treatment with the passive anti-A*β* immunotherapy AAB001 led to vasogenic edema in some individuals [2]. The A*β* immunogens that are now being used in the second generation of active immunization trials have been modified to reduce the risk of meningoencephalitis. However, academia, industry, and regulators are on their toes to identify immunological side effects as early as possible to avoid patient injury. Therefore, it is of utmost importance to identify and exclude patients who fulfill clinical AD criteria but who already before the start of therapy show biochemical signs of neuroinflammation. 

A number of standard laboratory tests are useful for this purpose. Typical laboratory findings in patients with neuroinflammatory/neuroinfectious conditions include impaired blood-brain barrier function, as reflected by an elevated ratio of albumin concentrations in cerebrospinal fluid (CSF) and serum/plasma, CSF monocytosis and IgG and IgM bands selectively in CSF [3, 4]. These changes are also seen in therapy-induced meningoencephalitis [1]. We here present two clinical AD cases who were diagnosed with Lyme neuroborreliosis (LNB) during screening for eligibility to enter an anti-A*β* immunotherapy trial. Should the two cases have been included and deteriorated, additional investigations might have led to the erroneous conclusion that therapy-induced meningoencephalitis had occurred.

## 2. Case Descriptions

NO is a 70-year-old retired man. His health history is remarkable for a cholecystectomy in 2001 that was followed by confusion. An epileptic seizure was suspected and the patient was treated with carbamazepine for a short period. He did not have any new seizures after carbamazepine was withdrawn. The patient has a positive family history of AD with both his grandfather and mother having been diagnosed with the disease.

In 1995, NO started to complain of cognitive decline that progressed over the years with aphasia, apraxia, and agnosia. He developed spatial deficits with orientation difficulties and a strong tendency to misplace belongings. In 2002, he was diagnosed with AD. A magnetic resonance imaging (MRI) scan of the brain was normal with no atrophy or white matter changes. EEG showed slow postcentral rhythm (7 Hz), and single positron emission computed tomography (SPECT) of the brain revealed frontotemporal hypoperfusion with left-side predominance. CSF analyses showed slightly elevated albumin ratio of 12 (<10) as a sign of mildly impaired blood-brain barrier function, normal cell counts, elevated total-tau (*T*-tau) of 589 ng/L (laboratory reference limit <400 ng/L), and normal A*β*1-42 concentration 495 ng/L (>450). He was heterozygous for the Alzheimer-associated *ε*4 allele of the apolipoprotein E gene. Neurological examination was normal, and the neuropsychological profile and clinical evaluation were consistent with AD. The patient started treatment with galantamine. His cognition improved with the minimental state examination (MMSE) score rising from 22 to 28 points. 

In November 2006, the patient was screened for a clinical trial of active immunization against A*β*. His cognition had been rather stable over the four years on treatment with only a minor decrease of the MMSE score from 28 to 25. He had no somatic complaints and his somatic status was normal. He showed no symptoms of infection and had no neurological complaints. An MRI scan of the brain showed slight atrophy with mild white matter changes consistent with AD. 

A lumbar puncture was performed at baseline before inclusion in the trial, and the biomarkers for AD, *T*-tau, phospho-tau_181_ (*P*-tau_181_), and A*β*1-42 were 520 (<400), 96 (<80), and 460 (>450) ng/L, respectively, which is consistent with AD. The albumin ratio was further elevated, and, surprisingly, CSF monocyte counts were high and so was the IgM-index (Table 1). Agarose gel electrophoresis of serum and CSF followed by immunoblotting against IgM supported ongoing intrathecal IgM production. This neurochemical picture is consistent with neuroinflammation. Anti-*Borrelia* titers were positive in CSF but negative in serum. The patient was treated with standard doxycycline 200 mg twice a day for 10 days. He was then followed with repeated CSF taps for 1.5 year during which his albumin ratio and cell counts were normalized. However, *T*-tau and *P*-tau181 remained pathologically elevated and A*β*1-42 turned low indicating an ongoing AD process. Accordingly, the patient has continued to deteriorate cognitively during the followup period. 

The next case, BS, is a 68-year-old woman. She is occupied as a hairdresser, still working part-time. Her health history is unremarkable. In 2003, she started to suffer from progressive cognitive deterioration, and in February 2006 she fulfilled clinical criteria for AD and started treatment with galantamine. The diagnosis was based on the anamnestic record and supported by findings on MRI and SPECT, as well as neuropsychological test results. To further secure her diagnosis, a CSF tap was planned but she declined this investigation. The patient was terrified of her diagnosis and wanted to fight it the best she could. She had heard of ongoing immunization trials at the Karolinska University Hospital and was referred for being evaluated for eligibility. As a part of this evaluation, a baseline CSF tap was mandatory and she agreed on this. The results showed completely normal *T*-tau, *P*-tau_181_, and A*β*1-42 concentrations in CSF speaking against AD. However, the albumin ratio was strongly elevated, CSF monocyte counts high, and both the IgG- and IgM-indices elevated (Table 1) with oligoclonal IgG and IgM bands selectively in CSF as a further sign of intrathecal IgG and IgM production. The anti-*Borrelia* titer in serum was negative but positive in CSF. She was treated with standard doxycycline 200 mg twice a day for 10 days. She was then followed with repeated CSF taps for 1.5 year showing normalization of the blood-brain barrier function and cell counts. Her cognitive status slowly improved during this period and the AD diagnosis was disposed.

## 3. Discussion

The two cases illustrate the necessity of identifying treatable causes of memory problems. The case histories are of relevance both to clinicians who evaluate and treat patients with cognitive problems and also to the pharmaceutical industry that designs trials of drug candidates that might impact the immune system. For one of the two cases, BS, the spinal tap was essential with the CSF test results radically changing the diagnosis from an incurable, ultimately fatal disease to a treatable spirochetal infection. NO suffers from AD but during the disease process he contracted LNB that could have been mistaken for a side effect of the anti-A*β* immunization should he have been included in the trial. 

LNB is a multisystem disease caused by infection with the spirochete *Borrelia burgdorferi* and the most common infectious cause of chronic neuroinflammation. Neurologic complications of LNB include encephalitis, meningitis, and dementia, as well as a broad range of peripheral neuropathies [5]. The many symptoms of LNB may mimic neurodegenerative diseases, such as AD and amyotrophic lateral sclerosis (ALS). Although there is no strong evidence that these diseases are caused by spirochetal infections, diagnosis of each must rule out the possibility of LNB. 

To the best of our knowledge, there are no published studies on the prevalence of LNB in patients attending a memory clinic. At least four studies have examined the prevalence of anti-*Borrelia burgdorferi* antibodies in serum in AD cases and controls without finding evidence of an association with AD [6–9]. Two cases of LNB were diagnosed in 1,089 consecutive patients (aged 23–89 years) who underwent lumbar puncture as a part of the routine diagnostic evaluation between February 2001 and March 2007 at the specialized memory clinic in Malmö [10], but it should be noted that these patients were selected referral patients. The prevalence of intrathecal immunoglobulin production in AD patients ranges from 5 to 20% in different studies [11–13]. 

In conclusion, analysis of CSF taken from patients at baseline, before treatment, could be useful in clinical trials for identifying and excluding individuals with chronic infectious or inflammatory CNS disorders that can mimic or aggravate the symptoms of AD. The inclusion of such cases in trials could result in the wrong conclusion that an adverse effect, such as encephalitis, was related to the drug being tested. Baseline CSF samples can also be used in comparisons with CSF collected after treatment initiation. The benefit of such comparisons is that even minor inflammatory activation within the CNS, as a result of adverse effects of the drug, can be identified. Thus, CSF biomarkers could allow safety monitoring during clinical trials. Longitudinal CSF sampling during the treatment period might indicate whether a certain drug induces harmful immune activation over the long term. 

Considering CSF analyses in a broader perspective, we think a spinal tap should be considered in every patient who seeks medical advice for cognitive problems, at least in areas where LNB is endemic and in particular if the clinical presentation is atypical. The safety and acceptability of the procedure support this view [10, 14, 15].

## Figures and Tables

**Table 1:** Biochemical findings in the cerebrospinal fluid of two patients with cognitive problems and Lyme neuroborreliosis.

Case	Albumin ratio (<10.2)	IgG-index (<0.63)	IgM-index (<0.060)	CSF monocytes (<5/*μ*L)	Serum anti-Borrelia antibodies	CSF anti-Borrelia antibodies
Man, 70 years	13.5	0.49	0.28	108	Negative	Positive
Woman, 68 years	33.4	1.82	0.93	84	Negative	Positive

Values in parentheses show laboratory reference limits.
